# Nicotinamide adenine dinucleotide metabolism and arterial stiffness after long-term nicotinamide mononucleotide supplementation: a randomized, double-blind, placebo-controlled trial

**DOI:** 10.1038/s41598-023-29787-3

**Published:** 2023-02-16

**Authors:** Takeshi Katayoshi, Sachi Uehata, Noe Nakashima, Takahisa Nakajo, Natsuko Kitajima, Masakatsu Kageyama, Kentaro Tsuji-Naito

**Affiliations:** DHC Corporation Laboratories, Division 2, 2-42 Hamada, Mihama-Ku, Chiba, 261-0025 Japan

**Keywords:** Biochemistry, Health care, Molecular medicine

## Abstract

Many animal studies have shown that oral administration of the nicotinamide adenine dinucleotide (NAD^+^) precursor nicotinamide mononucleotide (NMN) prevents the reduction of NAD^+^ levels in organs and tissues, helping alleviate aging-related diseases. However, there are very few clinical reports of NMN supplementation in humans. Thus, this study aimed to investigate the influence of a 12-week NMN oral supplementation on biochemical and metabolic health parameters. A 12-week randomized, double-blind, placebo-controlled, parallel-group clinical trial was conducted. A total of 36 healthy middle-aged participants received one capsule of either 125 mg NMN or placebo twice a day. Among the NAD^+^ metabolites, the levels of nicotinamide in the serum were significantly higher in the NMN intake group than in the placebo group. Pulse wave velocity values indicating arterial stiffness tended to decrease in the NMN intake group. However, no significant difference was found between the two groups. Long-term NMN supplementation at 250 mg/day was well tolerated and did not cause adverse events. NMN safely and effectively elevated NAD^+^ metabolism in healthy middle-aged adults. Additionally, NMN supplementation showed potential in alleviating arterial stiffness.

## Introduction

Nicotinamide adenine dinucleotide (NAD^+^) is a coenzyme for most redox reactions in metabolic pathways. In addition, NAD^+^ is a required co-substrate for several NAD^+^-consuming enzymes, such as sirtuins (SIRTs) and poly ADP-ribose polymerases (PARPs), that are involved in diverse biological processes. Since NAD^+^ levels decrease with age, and NAD^+^ metabolism deterioration exacerbates several age-related diseases, maintaining NAD^+^ levels is essential to preventing and treating them^1^. There are two main pathways for NAD^+^ biosynthesis in mammals: the de novo pathway from tryptophan and the salvage pathway consisting of precursors, such as nicotinamide (NAM), nicotinamide mononucleotide (NMN), and nicotinamide riboside (NR)^2^. Systemic NAD^+^ levels and nicotinamide phosphoribosyltransferase (NAMPT) activity, which serves the rate-limiting enzymatic step that converts NAM to NMN in the salvage pathway, have been found to decrease with age in various tissues^2^. Because NMN is a downstream product of the NAMPT reaction, NMN supplementation is assumed to increase NAD^+^ biosynthesis in a way that avoids the need for NAMPT. Thus, NMN has been shown to have beneficial effects, including SIRT1 activation, as an NAD^+^ booster in various animal models of age-related diseases^2,3^.

In animal studies, NMN administration effectively enhances NAD^+^ levels in various peripheral tissues under normal and pathophysiological conditions. In addition, evidence from recent research indicates that NMN has beneficial effects, including physiological function enhancement and therapeutic potential for diverse diseases. However, clinical trials on NMN supplementation in humans have only begun in the last few years^4^. In 2020, the first human clinical study evaluated the safety of single NMN oral administration^5^. Subsequently, repeated NMN intake increased the blood NAD^+^ levels in healthy participants^6,7^. Moreover, long-term NMN supplementation in amateur runners, prediabetic women, and older adults enhanced aerobic capacity, muscle insulin sensitivity, and sleep quality, respectively^8–10^.

Cardiovascular diseases (CVDs) are the heart and circulatory system disorders and are a major cause of morbidity and mortality in the elderly worldwide^11^. Metabolic disorders play a crucial role in CVDs, such as heart failure and atherosclerosis^12^. NAD^+^ is essential in metabolic pathways; therefore, supplementing NAD^+^ precursors is expected to improve these diseases by enhancing metabolic stability. In addition, the beneficial functions of SIRT1, an NAD^+^-dependent deacetylase, in cardiovascular aging are well documented at the preclinical level, supporting the idea that the enhanced NAD^+^ by administering its precursors may prevent CVDs^12^. In fact, several human clinical trials indicate that NR and NAM supplementation decrease the risk factors of CVDs, such as hypertension, arterial stiffness, and elevated level of low-density lipoprotein cholesterol^13,14^. However, although NMN supplementation improves age-associated vascular dysfunction in mice, its effects on human vascular function have not yet been validated. In this study, we conducted a double-blind, randomized, placebo-control clinical trial in middle-aged men and women. We evaluated the effects of 12-week NMN supplementation on blood NAD^+^ metabolites level, SIRT1 expression, and metabolic health parameters, including CVD risk factors.

## Results

### Baseline characteristics and completion of clinical study

**Figure 1:**
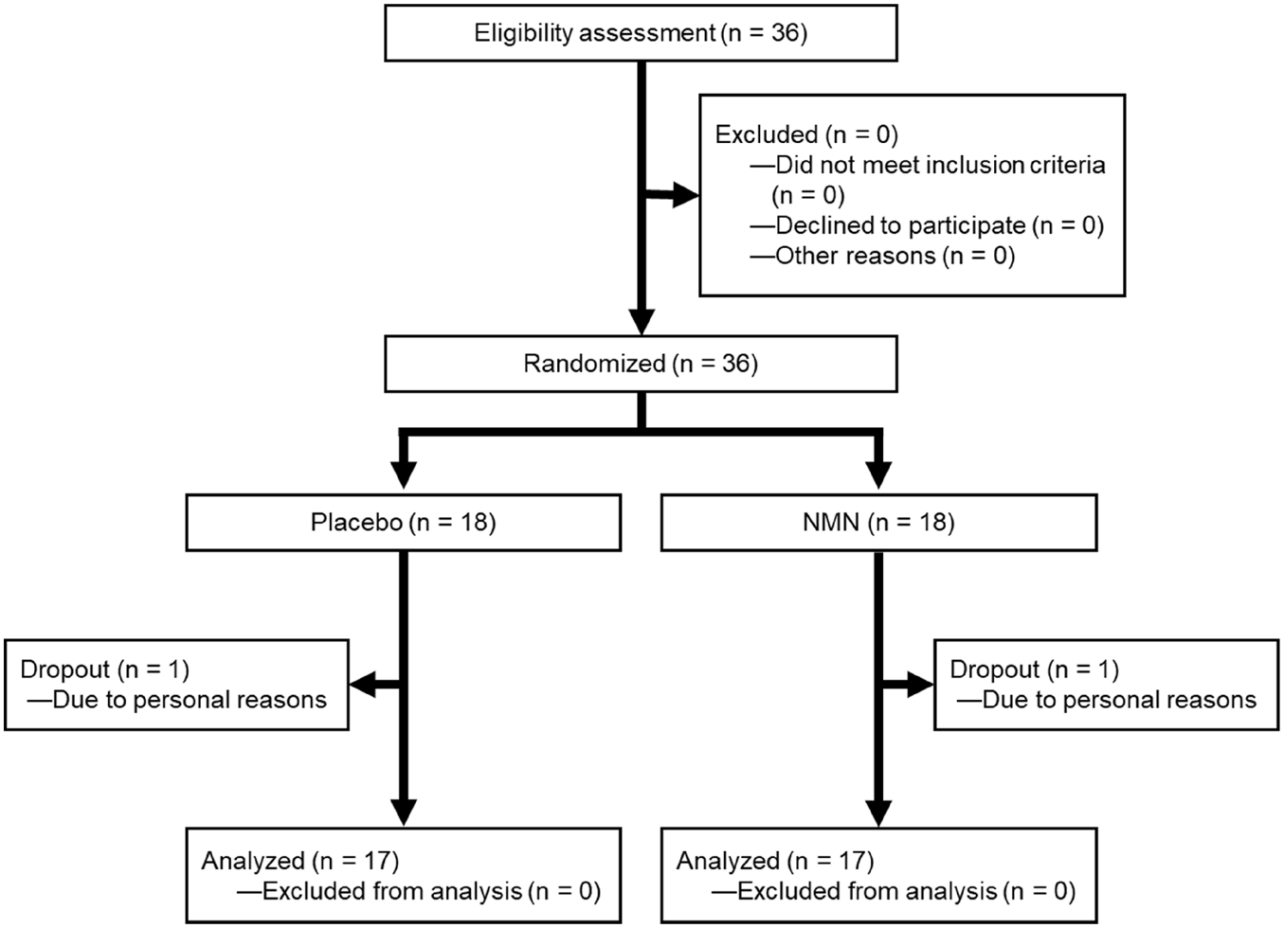
Clinical trial flowchart.

Figure 1 illustrates the study flowchart, which includes the processes of subject enrolment, allocation, and analysis. Thirty-six healthy men and women aged 40–59 years agreed to participate in the study. Subjects were randomly assigned to either the NMN intake (250 mg/day) or placebo group. The baseline characteristics of participants are shown in Table 1. At the eligibility assessment stage, no subjects were excluded, including those who did not meet the selection criteria, declined participation, or were excluded for other reasons. No significant differences were found between the groups in any baseline parameters (Table 1). Following the 12-week intervention, one participant in each group was untraceable and was considered a dropout. All subjects who completed the study showed no significant changes in parameters such as hematology, clinical chemistry, and hormones after the 12-week intervention (Table 2). These results indicate that long-term NMN supplementation at a dose of 250 mg/day is safe and well-tolerated in healthy middle-aged adults.

**Table 1:** Baseline characteristics of study participants.

	Placebo (n = 18)	NMN (n = 18)	P-value
Sex (Male/female)	6/12	8/10	0.733
Age (years)	47.9 ± 5.5	48.1 ± 5.4	0.951
Height (cm)	164.1 ± 6.2	167.7 ± 10.1	0.216
Weight (kg)	58.7 ± 9.1	62.9 ± 18.6	0.405
BMI (kg/M^2^)	21.7 ± 2.3	21.9 ± 4.3	0.829
Systolic blood pressure (mmHg)	124.2 ± 14.5	119.8 ± 17.5	0.419
Diastolic blood pressure (mmHg)	76.2 ± 12.0	71.7 ± 16.6	0.316

Values are presented as mean ± standard deviation. Each parameter was measured prior to the initiation of the intervention. To analyze any significant differences between groups, data on sex were assessed by the chi-square test, and other parameters such as age, weight, BMI, and blood pressure were tested using Welch’s *t* test. Statistical significance was set at *p* < 0.05.

*BMI* body mass index, *NMN* nicotinamide mononucleotide.

**Table 2:** Body composition and metabolic parameters of the placebo and NMN intake groups before and after the intervention period.

	Placebo (n = 17)			NMN (n = 17)			P-value
	Baseline	12 weeks	% change from baseline	Baseline	12 weeks	% change from baseline	
Weight (kg)	57.8 ± 8.4	57.6 ± 7.8	− 0.4	61.7 ± 18.5	61.9 ± 18.6	0.4	0.324
BMI (kg/M^2^)	21.5 ± 2.3	21.5 ± 2.2	0.0	21.7 ± 4.2	21.9 ± 4.3	0.8	0.273
Blood glucose level (mg/dL)	90.1 ± 5.9	89.9 ± 6.5	− 0.2	87.0 ± 4.5	89.0 ± 5.6	2.3	0.324
Blood count							
WBC (counts/µL)	6015.3 ± 1876.9	5999.4 ± 1732.2	− 0.3	5937.6 ± 1552.5	5853.5 ± 1871.5	− 1.4	0.838
RBC (counts × 10^4^//µL)	454.3 ± 42.7	442.1 ± 36.8	− 2.7	452.5 ± 41.9	445.8 ± 41.7	− 1.5	0.376
Hemoglobin (g/dL)	13.6 ± 1.0	13.4 ± 0.9	− 2.0	13.6 ± 1.8	13.3 ± 1.8	− 2.0	0.971
Hematocrit (%)	42.7 ± 2.9	41.6 ± 2.9	− 2.7	42.6 ± 4.1	41.7 ± 4.1	− 2.1	0.686
Blood pressure							
Systolic (mmHg)	124.3 ± 15.0	127.1 ± 13.0	2.2	118.0 ± 16.2	119.2 ± 18.2	1.0	0.414
Diastolic (mmHg)	76.0 ± 12.4	79.4 ± 10.3	4.4	69.8 ± 15.0	70.8 ± 15.5	1.5	0.196
Liver function							
AST (U/L)	25.3 ± 7.9	23.1 ± 6.1	− 8.6	22.8 ± 7.5	20.8 ± 5.4	− 9.0	0.472
ALT (U/L)	24.9 ± 12.2	22.4 ± 9.8	− 10.4	20.6 ± 10.9	17.4 ± 8.3	− 16.0	0.193
γ-GTP (U/L)	32.4 ± 19.2	29.6 ± 18.7	− 8.5	37.1 ± 46.2	33.0 ± 37.3	− 11.1	0.956
Lipids							
HDL-cholesterol (mg/dL)	73.5 ± 16.5	74.2 ± 16.9	1.0	72.2 ± 15.5	73.5 ± 16.2	1.7	0.885
LDL-cholesterol (mg/dL))	122.7 ± 54.1	121.3 ± 47.6	− 1.2	119.9 ± 25.9	120.3 ± 24.9	0.3	0.863
Triglyceride (mg/dL)	86.4 ± 54.7	83.1 ± 46.6	− 3.8	69.1 ± 35.9	70.6 ± 42.1	2.1	0.804
Hormones							
Testosterone (ng/dL)	143.1 ± 193.7	139.7 ± 187.8	− 2.4	212.2 ± 249.1	208.2 ± 257.6	− 1.9	0.933
Progesterone (ng/mL)	2.1 ± 4.4	3.0 ± 4.8	43.6	2.4 ± 6.2	1.9 ± 4.4	− 22.1	0.860
Estradiol (pg/mL)	75.2 ± 87.2	101.7 ± 123.8	35.1	81.0 ± 121.1	78.3 ± 82.4	− 3.3	0.361
DHEA-S (ng/mL)	1610.1 ± 566.1	1864.2 ± 751.2	15.8	1875.8 ± 1087.3	1923.1 ± 972.4	2.5	0.166
Serum cortisol (µg/dL)	8.4 ± 2.6	7.9 ± 2.5	− 6.6	8.1 ± 3.1	7.2 ± 1.9	− 11.0	0.422

Values are presented as mean ± standard deviation. Primary efficacy variables were statistically analyzed using a full-analysis-set population. Analysis of covariance (ANCOVA) was applied for significance between the two groups using the baseline as a covariate. Statistical significance was set at *p* < 0.05.

*NMN* nicotinamide mononucleotide, *BMI* body mass index, *WBC* white blood cell, *RBC* red blood cell, *AST* aspartate aminotransferase, *ALT* alanine aminotransferase, *γ-GTP* γ-glutamyl transpeptidase, *HDL* high-density lipoprotein, *LDL* low-density lipoprotein, *DHEA*-*S* dehydroepiandrosterone sulfate.

### Measurement of serum NMN, NAD^+^, and NAM

There have been no reports that NMN supplementation increases NMN blood levels. Therefore, to investigate whether the 12-week administration of NMN alters NMN levels in the blood and NAD^+^ metabolism, the serum concentrations of NMN, NAD^+^, and NAM were measured using isotope dilution liquid chromatography with tandem mass spectrometry analyses. As shown in Table 3, at baseline, serum NAM levels significantly differed between the placebo and NMN groups (*p* = 0.001). The analysis of covariance (ANCOVA) with baseline score as a covariate revealed that NAM levels in the NMN intake group were significantly increased after the intervention compared to the placebo group (*p* = 0.037). In addition, an intra-group comparison before and after the intervention revealed that serum NAM in the NMN group significantly increased after 12 weeks (*p* = 0.006), while that in the placebo group significantly decreased after intervention (*p* = 0.014). On the other hand, NAD^+^ and NMN in the serum were detectable in both groups, but the values were below the lower limit of quantification. Therefore, it was difficult to compare these two parameters between the groups. NAD^+^-consuming enzymes such as SIRTs and PARPs hydrolyze NAD^+^ to produce NAM and ADP-ribosyl products^2^. Thus, the increased NAM levels by NMN intervention indicate that NMN supplementation effectively enhanced NAD^+^ metabolism in middle-aged adults.

**Table 3:** NAD^+^ metabolites of the placebo and NMN intake groups before and after the intervention period.

	Placebo (n = 17)			NMN (n = 17)			P-value
	Baseline	12 weeks	% change from baseline	Baseline	12 weeks	% change from baseline	
NAM (ng/mL)	14.9 ± 3.8	10.9 ± 4.8	− 26.8	10.4 ± 3.5	16.5 ± 6.3	57.6	0.037*
NMN (ng/mL)	< 2	< 2	N.A	< 2	< 2	N.A	N.A
NAD (ng/mL)	< 5	< 5	N.A	< 5	< 5	N.A	N.A

Values are presented as mean ± standard deviation. Primary efficacy variables were statistically analyzed using a full-analysis-set population. Analysis of covariance (ANCOVA) was applied for significance between the two groups using the baseline as a covariate. Statistical significance was set at *p* < 0.05.

*NAM* nicotinamide, *NMN* nicotinamide mononucleotide, *NAD* nicotinamide adenine dinucleotide.

### Measurement of indicators related to blood vessel condition

**Figure 2:**
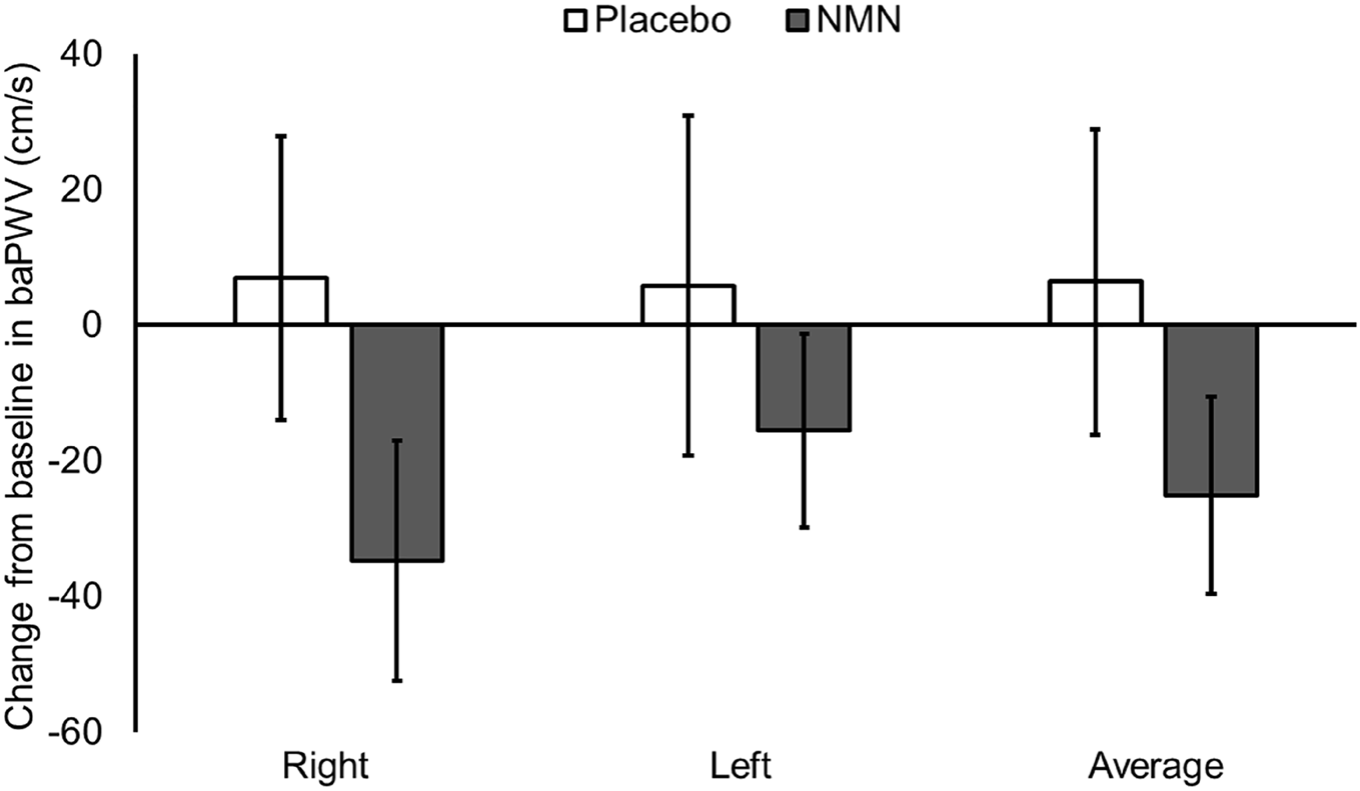
Change from baseline in brachial-ankle pulse wave velocity (baPWV) after nicotinamide mononucleotide (NMN) or placebo supplementation. Values are expressed as mean ± standard error.

We evaluated the effects of NMN supplementation on the conditions of blood vessels. In addition to biochemical and hematological tests, we measured the ankle-brachial index (ABI) and brachial-ankle pulse wave velocity (baPWV) to evaluate blood flow and arterial stiffness, respectively. There were no characteristic clinical findings in the blood pressure, number of blood cells, and ABI values in both groups (Tables 2 and 4). The average baPWV values in the NMN intake group tended to decrease by 25.1 ± 14.5 cm/s (Fig. 2). However, no significant difference was observed in the average baPWV values between the two groups (*p* = 0.097).

**Table 4:** Parameters for assessing the vascular function of the placebo and NMN intake groups before and after the intervention period.

	Placebo (n = 17)			NMN (n = 17)			P-value
	Baseline	12 weeks	% change from baseline	Baseline	12 weeks	% change from baseline	
ABI							
Right	1.11 ± 0.04	1.12 ± 0.06	1.3	1.10 ± 0.07	1.11 ± 0.07	0.6	0.623
Left	1.10 ± 0.06	1.13 ± 0.06	2.2	1.10 ± 0.07	1.11 ± 0.08	0.6	0.359
Average	1.11 ± 0.04	1.12 ± 0.06	1.8	1.10 ± 0.06	1.11 ± 0.06	0.6	0.367
BaPWV (cm/s)							
Right	1332.7 ± 227.1	1339.6 ± 217.1	0.5	1211.0 ± 153.8	1176.4 ± 161.7	− 2.9	0.070
Left	1343.6 ± 216.5	1349.4 ± 211.7	0.4	1205.1 ± 153.6	1189.5 ± 131.1	− 1.3	0.147
Average	1338.1 ± 219.9	1344.5 ± 212.9	0.5	1208.0 ± 152.2	1182.9 ± 145.3	− 2.1	0.097

Values are presented as mean ± standard deviation. Primary efficacy variables were statistically analyzed using a full-analysis-set population. Analysis of covariance (ANCOVA) was applied for significance between the two groups using the baseline as a covariate. Statistical significance was set at *p* < 0.05.

*ABI* ankle-brachial index, *baPWV* brachial-ankle pulse wave velocity, *NMN* nicotinamide mononucleotide.

**Figure 3:**
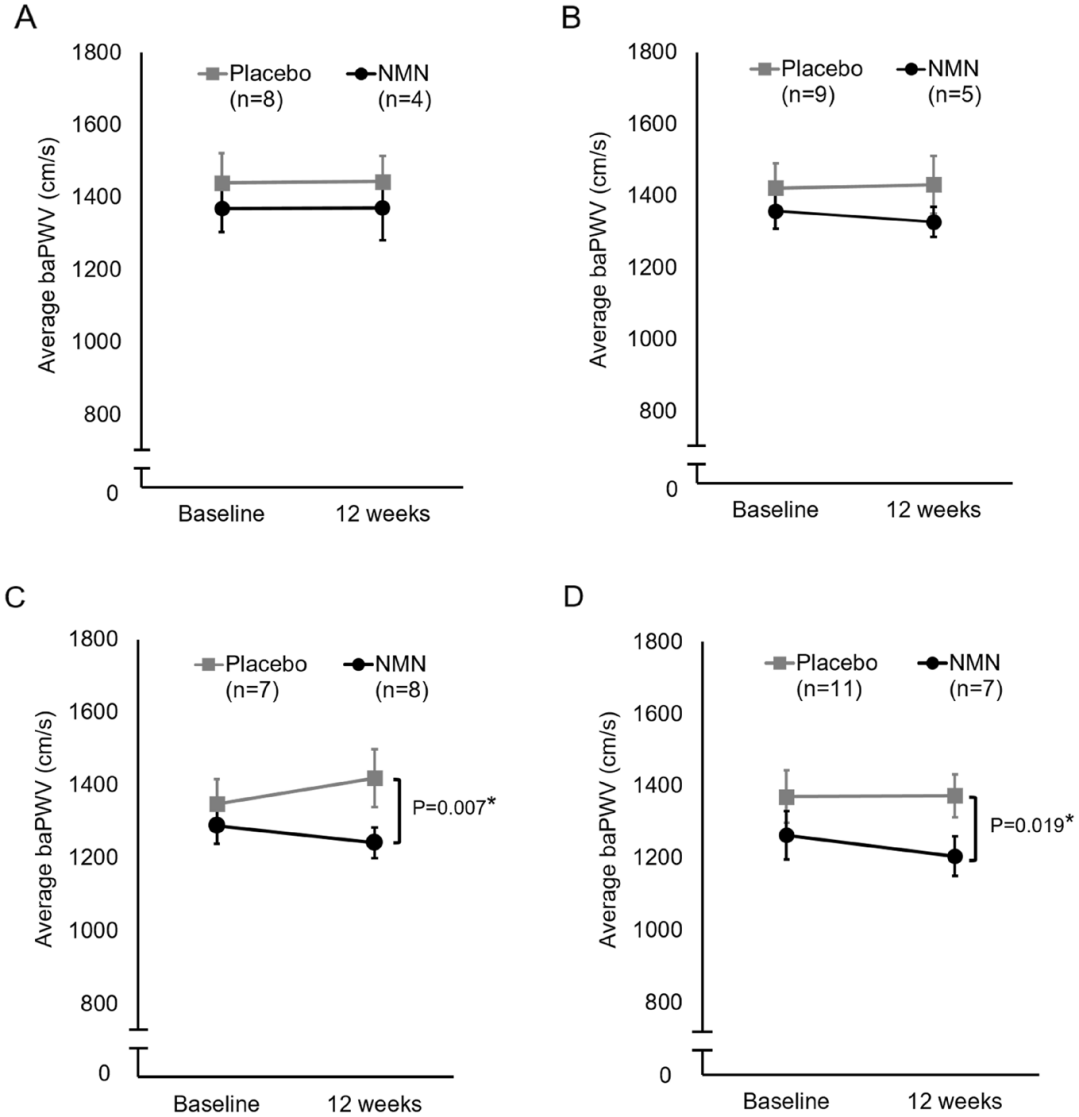
Subgroup analyses of NMN and placebo groups for baPWV. (**A**) Subjects with above-average systolic blood pressure, (**B**) subjects with above-average diastolic blood pressure, (**C**) subjects with above-average BMI, and (**D**) subjects with above-average blood glucose levels. Values are expressed as mean ± standard error.

Since hypertension, obesity, and hyperglycemia are considered cardiovascular risk factors, we performed subgroup analyses of NMN and placebo groups in subjects with above-average blood pressure, body mass index (BMI), or blood glucose level. In the analyses of subjects with higher-than-mean systolic or diastolic blood pressure, there was no significant change in baPWV values between the groups during the test period (Fig. 3A,B). In contrast, in subjects with above-average BMI or blood glucose levels, baPWV values in the NMN intake group were significantly decreased after the test period compared to the placebo group (Fig. 3C,D). These results suggest that NMN supplementation potentially improves vascular health in middle-aged adults, especially in those with high BMI or blood glucose levels.

### Measurements of other health parameters

We assessed the effects of NMN administration on three health care parameters: urinary 8-hydroxydeoxyguanosine (8-OHdG), SIRT1 mRNA expression in the blood, and advanced glycation end products (AGEs) in the skin. However, no significant differences were observed between the two groups (Table 5).

**Table 5:** Health parameters of the placebo and the NMN intake groups before and after the intervention period.

	Placebo (n = 17)			NMN (n = 17)			P-value
	Baseline	12 weeks	% change from baseline	Baseline	12 weeks	% change from baseline	
SIRT1 mRNA level	3.6 ± 1.0	4.9 ± 1.4	38.5	3.6 ± 1.6	4.3 ± 2.0	18.8	0.211
AGEs (a.u.)	0.6 ± 0.1	0.5 ± 0.1	− 3.9	0.5 ± 0.1	0.5 ± 0.1	7.6	0.283
8-OHdG (ng/mg creatinine)	7.1 ± 2.4	7.3 ± 2.6	2.3	7.9 ± 2.6	8.0 ± 3.3	0.7	0.735

Values are presented as mean ± standard deviation. Primary efficacy variables were statistically analyzed using a full-analysis-set population. Analysis of covariance (ANCOVA) was applied for significance between the two groups using the baseline as a covariate. Statistical significance was set at *p* < 0.05.

*SIRT1* sirtuin 1, *AGEs* advanced glycation end products, 8-*OHdG* 8-hydroxydeoxyguanosine, *NMN* nicotinamide mononucleotide.

### Comparison of other supplements consumed during the study

During the study, the percentage of people consuming other supplements in the placebo and NMN groups was 35.1% and 32.4%, respectively. Eight supplements (multivitamins, multiminerals, soy isoflavone, vitamin C, vitamin B complex, omega-3 fatty acids, lutein, and coenzyme Q10) were taken by over 10% of the total participants. Among them, the placebo group tended to consume more vitamin C, vitamin B complex, and omega-3 fatty acid supplements than the NMN group: N (placebo/NMN) = 4/2, 4/1, and 5/0, respectively. All the supplements consumed during the study are presented in Supplementary Table S1.

## Discussion

In this study, NMN supplementation was shown to be safe and beneficial for the activation of NAD metabolism in apparently healthy middle-aged individuals. We also found that 12-week supplementation with 250 mg NMN tended to reduce baPWV values. A previous meta-analysis of longitudinal cohort studies investigating baPWV showed that the risk of cardiovascular events associated with high baPWV is almost three times that associated with low baPWV^15^. Thus, NMN supplementation may alleviate vascular stiffness and reduce the risk of CVD events. One clinical trial report has reported similar results on another NAD^+^ precursor NR^13^. In that study, a 6-week supplementation with 1000 mg/day of NR tended to reduce carotid-femoral PWV in healthy middle-aged and older people^13^. These two studies, including ours, did not reveal significant differences in PWV data; however, they suggest that chronic supplementation of NAD^+^-boosting agents may reduce blood vessel stiffness, thereby protecting against CVD risk.

Indicators of arterial stiffness, such as PWV, have been reported to correlate well with age, and the estimated age of blood vessels can be calculated from a subject’s baPWV value^16^. The change in the estimated age of blood vessels in the placebo and NMN intake groups was calculated using the equation reported by Tomiyama and was 0.5 ± 6.1 and − 2.0 ± 4.3, respectively. In addition to aging, sex differences are another aspect to consider in arterial stiffness. The baPWV value is lower in women than in men until age 60^16^. Accordingly, subgroup analyses were performed by categorizing the participants as men and women. The baseline average baPWV values in women were lower by 52.2 cm/s than in men. However, in both male and female subgroups, there were no differences between the placebo and NMN groups after the 12-week supplementation in baPWV values and other parameters, including ABI, blood pressure, BMI, and blood glucose level (data not shown).

As observed in the subgroup analyses limited to participants with higher-than-mean CVD risk factors, NMN’s baPWV-reducing effect was more pronounced in participants with higher BMI and blood glucose levels. Interestingly, these subgroups had no change in BMI and blood glucose levels after the 12-week supplementation. However, NMN intervention significantly decreased diastolic blood pressure in participants with higher-than-mean blood glucose levels (Supplementary Table S2). Hypertension is a known risk factor for CVDs. It has been clarified that a relatively mild increase in blood pressure increases cardiovascular risk even when the blood pressure is within a range that is considered healthy^17^. Thus, from the viewpoint of CVD prevention, it is important to control blood pressure at any stage, even if it falls within the healthy range. Vidal-Petiot et al. showed that a diastolic blood pressure of 80–89 mmHg increased CVD risk compared with 70–79 mmHg, while a systolic blood pressure of 130–139 mmHg did not, and concluded that it is more significant for patients with coronary artery disease to prioritize diastolic blood pressure < 80 mmHg as a blood pressure-lowering target than systolic blood pressure < 130 mmHg^18^. Therefore, as observed in the subgroup analysis, the ameliorating effect of NMN supplementation on diastolic blood pressure may help reduce CVD risk, even in people who seem to be healthy.

In recent years, it has been clarified that adipocytokines secreted by adipocytes are closely related to CVDs^19^, and clinical studies have also reported a significant association between decreased serum adiponectin and increased PWV^20^. In vivo studies have reported that deficiency of the NAMPT gene leads to reduced adiponectin production, while oral NMN intake normalizes it^21^. Thus, restoration of adiponectin production may be a reasonable mechanism by which NMN improves PWV. In addition, a previous animal study showed that oral NMN ingestion reverses vascular dysfunction and increased aortic PWV by decreasing oxidative stress in old mice^3^. Hence, investigating how NMN reduces endothelial oxidative stress may provide a better understanding of its underlying molecular mechanism.

This study’s results may have been influenced by several limitations: inappropriate recruitment of participants, the significant differences between both groups in baseline parameters other than those detailed in Table 1, and the inadequate sampling method to assess the NAD^+^ metabolism. We did not set strict healthy ranges for the baseline characteristics of the participants, which may have influenced evaluating NMN’s beneficial effects in healthy middle-aged adults. In addition, the baseline baPWV values (right, left, and average) in the NMN group were lower than in the placebo group, and especially the left baPWV values significantly differed between the placebo and NMN intake groups. NAD^+^ and its metabolites were analyzed using serum samples rather than whole or peripheral blood mononuclear cells. The primary purpose of those analyses was to assess the extracellular NMN levels prior to being taken up by blood cells and converted to NAD^+^. However, NAD^+^ and its metabolites are prominent in the non-serum fraction. Thus serum sampling was considered unsuitable for their quantification. In addition, since supplements that participants had taken prior to this study were not excluded, other supplements differed in consumption between both groups and may have influenced the results. Especially, omega-3 fatty acids, observed only in the placebo group, may have at least influenced the parameters related to vascular health because it improves vascular function and reduces CVD risk factors^22,23^. Therefore, it seems better to consider these points when conducting additional tests in the future.

In conclusion, this study provides the first insight into the effects of long-term NMN supplementation on CVD risk factors, including baPWV value, suggesting that further clinical trials are beneficial to establishing NMN’s benefits for reducing arterial stiffness.

## Materials and methods

### Study design

Thirty-six healthy male and female subjects were enrolled in this randomized, double-blind, placebo-controlled, parallel study. Written informed consent was obtained from all subjects involved in the study. Subjects were randomly assigned to two groups by Orthomedico Inc. (Tokyo, Japan). Subjects visited the clinic in Tokyo, Japan, for laboratory tests and safety assessments at baseline prior to the initiation of the intervention. All results were reviewed by a physician. Subjects were provided with either NMN (125 mg/capsule) or placebo capsules in batches for 12 weeks, and one capsule was ingested twice a day after meals for 12 weeks. NMN capsules contained NMN, cellulose, silicon dioxide, and calcium stearate. In contrast, placebo capsules contained cornstarch, cellulose, silicon dioxide, and calcium stearate. After the 12-week intervention period, subjects returned to the clinic for laboratory tests and safety assessment. All the subjects were recruited between June 22 and July 7, 2021, and the last subject’s visit was on December 8, 2021. The study was completed after the last subject completed the last visit. The researchers involved in collecting and analyzing the results were not informed of the treatment conditions. We did not perform a follow-up analysis after terminating NMN supplementation. All the procedures in this study were approved by the DHC ethics committee on 04/08/2021 (No.202101). This study was conducted in accordance with the ethical principles that have their origins in the Declaration of Helsinki and its subsequent amendments, and was registered with the identifier UMIN000045205 on 20/08/2021 in the clinical trial registration system of the University Hospital Medical Information Network and met the criteria of the International Committee of Medical Journal Editors.

### Subjects

Eligible subjects were men and non-pregnant or non-breastfeeding women between 40 and 65 years of age who seemed healthy at the time of provision of consent to participate in the study. The key exclusion criteria were as follows: (1) history of serious hepatic, renal, cardiac, pulmonary, or gastrointestinal (including gastrectomy) disease; diabetes, food allergies, or other serious comorbidities; (2) use of drugs that may affect the test values in this study; and (3) participation in other clinical trials. Subjects continued to take dietary supplements or medicines that they had taken prior to the study and avoided starting any new dietary supplements during the study.

### Randomization and blinding

An allocation controller at Orthomedico Inc. (Tokyo, Japan) randomly assigned subjects to two supplementation groups (NMN or placebo) by block random allocation. The allocation ratio of each group was 1:1. An allocation table was created based on the algorithm of block random allocation. The allocation table contained the types of groups (NMN or placebo) and the identification code of each individual and was kept concealed using an emergency key by the allocation controller until the blinding review was completed. DHC Corporation provided the NMN and placebo capsules to the allocation controller. After making sure that the capsules were indistinguishable, the allocation controller assigned an identification code to all the capsules based on the allocation table. After the screening test, the capsules with the identification code was to subjects by a blinded investigator. Information on allocation was not opened until the subjects for analysis were determined at a clinical meeting after test completion.

### Characteristics of subjects

BMI (kg/m^2^) was calculated as weight divided by height squared. Systolic and diastolic blood pressure were measured using a Heart Station S MPV-550 (A&D, Tokyo, Japan). Blood and urine samples were collected at the clinic, and subsequent tests investigating blood glucose levels, liver function, lipids, and hormones were performed by outsourcing their analyses to BML (Tokyo, Japan). ABI and baPWV were measured using a waveform analyzer (BP-203RPE III, Omron, Kyoto, Japan).

### Quantification of serum NAD^+^, NMN, and NAM

The serum concentrations of NAM, NMN, and NAD^+^ were calculated by outsourcing their analyses to LSI Medience (Tokyo, Japan). Briefly, the serum was transferred to a test tube and mixed with methanol (1/6, v/v). After being centrifuged at 1000×*g* for 15 min, the supernatant was dried in nitrogen gas at 40 °C. The residue was dissolved in a 5 mM ammonium acetate solution and analyzed using a liquid chromatography-tandem mass spectrometer (6470 B Triple Quadrupole system, Agilent, CA, US) equipped with a reverse-phase Aqcuity HSS T3 column (2.0 × 100 mm, 1.8 µm, Waters, MA, US). Quantitative analyses were conducted using Mass Hunter software (Agilent). The peak area was corrected using an internal standard, and the concentration of each component was determined using a calibration curve.

### Real-time PCR analysis of SIRT1 mRNA expression

SIRT1 mRNA expression in the blood was evaluated by outsourcing the analysis to the Research Center for Immunological Analysis (Okayama, Japan). Briefly, whole blood was collected in a PAXgene RNA blood collection tube (Qiagen, Mississauga, Canada). Total RNA was isolated from the blood using a PAXgene Blood RNA Kit (Qiagen) and stored in RNase-free water at − 80 °C. First-strand cDNA was reverse-transcribed with total RNA using a T100 Thermal Cycler (Bio-Rad, CA, USA). The mRNA expression level of the target genes was measured using a CFX384 Touch Real-Time PCR Detection System (Bio-Rad). *β-Actin* was used as a housekeeping gene for normalization. The comparative C_T_ method was used to calculate the relative levels of SIRT1 mRNA.

### Measurement of urine 8-OHdG

Urine 8-OHdG content was measured by outsourcing the analysis to the Nikken Seil (Shizuoka, Japan). Briefly, urine 8-OHdG was quantified using an enzyme-linked immunosorbent assay kit for 8-OHdG (Nikken Seil), according to the manufacturer’s instructions. Measured urinary 8-OHdG levels were normalized to urinary creatinine concentrations and are shown as the urinary 8-OHdG (ng/mL)/creatinine (mg/mL) ratio.

### Measurement of AGEs

AGEs were measured on the middle finger using an AGE sensor RQ-1201 J (Sharp Life Science, Kobe, Japan), according to the manufacturer’s instructions. Excitation light from the light source through the lens illuminates the skin surface of the fingertip inserted into the sensor. The emission spectrum reflected from the skin is detected as the accumulation of AGEs in the skin. The value of AGEs obtained with this device has been reported to correlate with the blood levels of methylglyoxal-derived hydroimidazolone-1, a major AGE in proteins^24^.

### Evaluation of safety

Participants were allowed to immediately inform the investigator and discontinue the capsules if they had any difficulty or challenge during the study. Adverse events were monitored by physician interviews and blood tests and surveyed using questionnaires after the 12-week intervention.

### Outcomes

The primary objectives of this study were to evaluate the efficacy of NMN supplementation on serum concentrations of NAD^+^, NMN, and NAM and SIRT1 expression levels in middle-aged participants after 12 weeks. The study’s secondary objectives were to assess the safety and health parameters, including blood count, blood pressure, liver function, lipids, hormones, AGEs, 8-OHdG levels, and vascular stiffness.

### Statistical analyses

The sample size was determined by reference to previous publications with primary trial objectives similar to our study^6,7^. Statistical analyses of the baseline characteristics of subjects were performed in an intention-to-treat population. For analyzing significant differences between groups, data on sex were assessed by the chi-square test, and other parameters, such as age, height, weight, BMI, blood pressure, and serum NAM were tested using Welch’s t-test. The primary efficacy variables were statistically analyzed using a full-analysis-set population. The ANCOVA was applied to the significance between groups using the baseline as a covariate. The paired *t*-test was used to compare the NAM levels pre- and post-intervention in each group. Statistical analyses were performed using IBM SPSS Statistics version 23 software (IBM, NY, USA). Statistical significance was set at *p* < 0.05.

## Supplementary Information

Supplementary Table S1.

Supplementary Table S2.

## Data Availability

The datasets generated during and/or analyzed during the current study are available on request. Contact: knaito@dhc.co.jp.
