# Supplementary material for: Nicotinamide adenine dinucleotide metabolism and arterial stiffness after long-term nicotinamide mononucleotide supplementation: a randomized, double-blind, placebo-controlled trial
Source: Sci Rep. 2023 Feb 16;13:2786. doi: 10.1038/s41598-023-29787-3 (PMC9935856; doi:10.1038/s41598-023-29787-3)
Supplement: Supplementary file 1 — Supplementary Table S1. [file 41598_2023_29787_MOESM1_ESM.docx]

**Supplementary Table S1. Other supplements consumed during the study**

| Type of supplements | Placebo (n= 17) | NMN (n= 17) | Total (n= 34) |
| --- | --- | --- | --- |
| Multivitamins | 4 | 5 | 9 |
| Multiminerals | 5 | 4 | 9 |
| Soy isoflavone | 4 | 4 | 8 |
| Vitamin C | 4 | 2 | 6 |
| Vitamin B complex | 4 | 1 | 5 |
| Omega-3 fatty acids | 5 | 0 | 5 |
| Lutein | 2 | 2 | 4 |
| Coenzyme Q10 | 2 | 2 | 4 |
| Vitamin D | 1 | 2 | 3 |
| Calcium | 1 | 2 | 3 |
| Lactic acid bacteria Bifidobacterium | 1 | 2 | 3 |
| Bilberry | 1 | 2 | 3 |
| Turmeric | 2 | 1 | 3 |
| Heme iron | 2 | 1 | 3 |
| Placenta | 3 | 0 | 3 |
| Red yeast rice | 3 | 0 | 3 |
| Vitamin E | 2 | 0 | 2 |
| Zinc | 1 | 1 | 2 |
| Astaxanthin | 1 | 1 | 2 |
| Propolis | 0 | 2 | 2 |
| Collagen | 1 | 0 | 1 |
| Citrulline | 0 | 1 | 1 |
| Theanine | 0 | 1 | 1 |
| Hatomugi | 0 | 1 | 1 |
| Pycnogenol | 0 | 1 | 1 |
| Saw palmetto extract | 0 | 1 | 1 |
| L-cysteine | 1 | 0 | 1 |
| Force collie | 1 | 0 | 1 |
| 3-hydroxy-3-methylbutyrate | 1 | 0 | 1 |

Values are presented as number of subjects.
