# Supplementary material for: Nicotinamide adenine dinucleotide metabolism and arterial stiffness after long-term nicotinamide mononucleotide supplementation: a randomized, double-blind, placebo-controlled trial
Source: Sci Rep. 2023 Feb 16;13:2786. doi: 10.1038/s41598-023-29787-3 (PMC9935856; doi:10.1038/s41598-023-29787-3)
Supplement: Supplementary file 2 — Supplementary Table S2. [file 41598_2023_29787_MOESM2_ESM.docx]

**Supplementary Table S2. Metabolic function, vascular function, and health parameters in subjects with above-average blood glucose level**

|  | Placebo (n = 11) | | |  | NMN (n = 7) | | | P-value |
| --- | --- | --- | --- | --- | --- | --- | --- | --- |
|  | Baseline | 12 weeks | % Change from  baseline |  | Baseline | 12 weeks | % Change from  baseline |  |
| Weight (kg) | 61.1 ± 7.2 | 60.8 ± 6.8 | -0.6 |  | 71.6 ± 22.8 | 71.3 ± 23.0 | -0.5 | 0.947 |
| BMI (kg/M^2^) | 22.5 ± 2.0 | 22.5 ± 2.0 | -0.2 |  | 23.9 ± 5.3 | 24.0 ± 5.5 | 0.2 | 0.765 |
| Blood glucose level (mg/dL) | 93.2 ± 4.2 | 93.1 ± 5.2 | -0.1 |  | 91.4 ± 2.0 | 92.6 ± 5.3 | 1.3 | 0.750 |
| Blood count |  |  |  |  |  |  |  |  |
| WBC (counts/µL) | 6049.1 ± 1764.3 | 6174.5 ± 1569.9 | 2.1 |  | 5357.1 ± 1470.0 | 5524.3 ± 1604.5 | 3.1 | 0.881 |
| RBC (counts×10^4^//µL) | 464.2± 25.8 | 450.1 ± 23.5 | -3.0 |  | 454.4 ± 53.2 | 452.9 ± 55.4 | -0.5 | 0.276 |
| Hemoglobin (g/dL) | 14.0 ± 0.9 | 13.7 ± 0.8 | -2.1 |  | 14.1 ± 1.6 | 14.0 ± 1.8 | -0.5 | 0.455 |
| Hematocrit (%) | 43.8 ± 2.3 | 42.3 ± 2.6 | -3.4 |  | 43.3 ± 3.7 | 43.2 ± 4.0 | -0.4 | 0.201 |
| Blood pressure |  |  |  |  |  |  |  |  |
| Systolic (mmHg) | 124.5 ± 12.2 | 127.6 ± 8.7 | 2.6 |  | 123.6 ± 18.6 | 123.9 ± 19.2 | 0.2 | 0.333 |
| Diastolic (mmHg) | 77.1 ± 11.0 | 82.1 ± 7.9 | 6.5 |  | 75.3 ± 15.7 | 72.6 ± 18.5 | -3.6 | 0.016* |
| Liver function |  |  |  |  |  |  |  |  |
| AST (U/L) | 24.5 ± 6.1 | 22.8 ± 5.2 | -7.0 |  | 23.6 ± 3.2 | 23.4 ± 5.6 | -0.6 | 0.504 |
| ALT (U/L) | 26.5 ± 12.4 | 22.9 ± 9.5 | -13.4 |  | 27.3 ± 12.1 | 23.9 ± 8.3 | -12.6 | 0.856 |
| γ-GTP (U/L) | 37.3 ± 19.9 | 31.5 ± 15.4 | -15.4 |  | 57.6 ± 66.8 | 51.6 ± 52.7 | -10.4 | 0.463 |
| Lipids |  |  |  |  |  |  |  |  |
| HDL-cholesterol (mg/dL) | 68.5 ± 13.3 | 67.5 ± 12.0 | -1.3 |  | 61.9 ± 16.1 | 63.4 ± 15.3 | 2.5 | 0.735 |
| LDL-cholesterol (mg/dL) | 132.0 ± 45.1 | 129.9 ± 37.4 | -1.6 |  | 134.3 ± 19.9 | 130.3 ± 20.1 | -3.0 | 0.922 |
| Triglyceride (mg/dL) | 103.4 ± 60.1 | 98.7 ± 47.0 | -4.5 |  | 79.0 ±43.3 | 84.7 ± 54.3 | 7.2 | 0.697 |
| Hormones |  |  |  |  |  |  |  |  |
| Testosterone (ng/dL) | 206.6 ± 217.8 | 197.5 ± 214.3 | -4.4 |  | 339.5 ± 232.2 | 317.9 ± 241.2 | -6.4 | 0.978 |
| Progesterone (ng/mL) | 0.7 ± 2.2 | 3.2 ± 5.5 | 353.2 |  | 0.4 ± 0.9 | 2.1 ± 5.6 | 417.2 | 0.814 |
| Estradiol (pg/mL) | 69.2 ± 95.5 | 71.2 ± 93.9 | 2.9 |  | 18.8 ± 11.4 | 28.3 ± 37.5 | 50.4 | 0.682 |
| DHEA-S (ng/mL) | 1565.9 ± 554.6 | 1803.7 ± 748.4 | 15.2 |  | 1930.3 ± 1058.2 | 2021.1 ± 949.9 | 4.7 | 0.594 |
| Serum cortisol (µg/dL) | 9.2 ± 2.2 | 8.0 ± 2.7 | -12.9 |  | 7.9 ± 2.5 | 8.3 ± 1.1 | 4.9 | 0.619 |
| ABI |  |  |  |  |  |  |  |  |
| Right | 1.10 ± 0.04 | 1.11 ± 0.07 | 1.2 |  | 1.10 ± 0.04 | 1.14 ± 0.05 | 3.4 | 0.409 |
| Left | 1.09 ± 0.06 | 1.13 ± 0.07 | 3.6 |  | 1.08 ± 0.10 | 1.13 ± 0.06 | 4.2 | 0.994 |
| Average | 1.10 ± 0.04 | 1.12 ± 0.06 | 2.4 |  | 1.09± 0.06 | 1.13 ± 0.06 | 3.8 | 0.549 |
| BaPWV (cm/s) |  |  |  |  |  |  |  |  |
| Right | 1374.7 ± 251.3 | 1377.5 ± 209.6 | 0.2 |  | 1270.0 ± 173.3 | 1194.0 ± 156.2 | -6.0 | 0.013* |
| Left | 1366.0 ± 239.3 | 1367.8 ± 189.3 | 0.1 |  | 1255.4 ± 187.1 | 1214.6 ± 136.1 | -3.3 | 0.291 |
| Average | 1370.4 ± 243.3 | 1372.6 ± 198.6 | 0.2 |  | 1262.7 ± 178.7 | 1204.3 ± 144.8 | -4.6 | 0.019* |
| SIRT1 mRNA level | 3.3 ± 1.0 | 4.5 ± 1.2 | 34.7 |  | 3.2 ± 0.8 | 4.1 ± 1.2 | 27.8 | 0.462 |
| AGEs (a.u.) | 0.5 ± 0.1 | 0.5 ± 0.1 | 0.9 |  | 0.5 ± 0.1 | 0.5 ± 0.0 | 0.8 | 0.978 |
| 8-OHdG (ng/mg creatinine) | 7.6 ± 2.6 | 7.0 ± 3.1 | -7.4 |  | 7.8 ± 1.3 | 7.7 ± 2.0 | -1.6 | 0.641 |

Values are presented as mean ± standard deviation. Analysis of covariance was applied for significance between the groups using the baseline as a covariate. Statistical significance was set at *p* < 0.05. NMN, nicotinamide mononucleotide; BMI, body mass index; WBC, white blood cell; RBC, red blood cell; AST, aspartate aminotransferase; ALT, alanine aminotransferase; γ-GTP, γ-glutamyl transpeptidase; HDL, high-density lipoprotein; LDL, low-density lipoprotein; DHEA-S, dehydroepiandrosterone sulfate; ABI, ankle-brachial index; baPWV, brachial-ankle pulse wave velocity; SIRT1, sirtuin 1; AGEs, advanced glycation end products; 8-OHdG, 8-hydroxydeoxyguanosine.
